# HSV-2 Co-Infection as a Driver of HIV Transmission among Heterosexual Non-Injecting Drug Users in New York City

**DOI:** 10.1371/journal.pone.0087993

**Published:** 2014-01-31

**Authors:** Don C. Des Jarlais, Kamyar Arasteh, Courtney McKnight, David C. Perlman, Jonathan Feelemyer, Holly Hagan, Hannah L. F. Cooper

**Affiliations:** 1 The Baron Edmond de Rothschild Chemical Dependency Institute, Beth Israel Medical Center, New York, New York, United States of America; 2 School of Nursing, New York University, New York, New York, United States of America; 3 Rollins School of Public Health, Emory University, Atlanta, Georgia, United States of America; University of Missouri-Kansas City, United States of America

## Abstract

**Objective:**

To examine herpes simplex virus 2 (HSV-2)/HIV co-infection as a contributing factor in the increase in HIV infection among non-injecting heroin and cocaine users in New York City.

**Methods:**

Subjects were recruited from the Beth Israel Medical Center drug detoxification and methadone maintenance programs in New York City in 1995–1999 and 2005–2011. All reported current heroin and/or cocaine use and no injection drug use. A structured questionnaire was administered and serum samples collected for HIV and HSV-2 testing. Population-attributable risk percentages (PAR%s) were estimated for associations between HSV-2 and increased susceptibility to and increased transmissibility of HIV among female NIDUs.

**Results:**

785 subjects were recruited from 1995–1999, and 1764 subjects from 2005–2011. HIV prevalence increased from 7% to 13%, with nearly uniform increases among all demographic subgroups. HSV-2/HIV co-infection was common in both time periods, with an average (over the two time periods) of 80% of HIV negative females infected with HSV-2, an average of 43% of HIV negative males infected with HSV-2; an average of 97% of HIV positive females also infected with HSV-2 and an average of 67% of HIV positive males also infected with HSV-2. The increase in HIV prevalence was predominantly an increase in HSV-2/HIV co-infection, with relatively little HIV mono-infection in either time period. The estimated PAR%s indicate that approximately half of HIV acquisition among females was caused by HSV-2 infection and approximately 60% of HIV transmission from females was due to HSV-2 co-infection.

**Conclusions:**

The increase in HIV infection among these non-injecting drug users is better considered as an increase in HSV-2/HIV co-infection rather than simply an increase in HIV prevalence. Additional interventions (such as treatment as prevention and suppressing the effects of HSV-2 on HIV transmission) are needed to reduce further HIV transmission from HSV-2/HIV co-infected non-injecting drug users.

## Introduction

While injecting drug use is strongly associated with HIV in many parts of the world. [1], non-injecting drug use may lead to sexual transmission of HIV through several pathways. Non-injecting drug use can lead to HIV transmission through impaired decision making while under the influence of drugs, or through increased sexual pleasure (from some drugs) leading to increased unsafe sexual activity, and through unsafe sex as part of exchanging sex for drugs or money to purchase drugs. [2]; [3]; [4] A number of studies have found very high HIV prevalence among (predominantly heterosexual) non-injecting drug users (NIDUs): 37% in Porto Alegre, Brazil [5], 43% in China [6], 13% in Canada [7], 20% in Florida [8], 19% in New York City [9], 24% in Portugal [10], and 29% in Russia [11].

In this report, we examine the relationships between HSV-2 and HIV infection over a time period in which HIV prevalence doubled among non-injecting drug users in New York City. HSV-2 increases susceptibility to HIV infection through physical disruption of the epithelial surface by HSV-2, and recruitment and persistence of inflammatory cells in the genital tract during HSV-2 reactivation at mucosal surfaces, [12]; [13]; [14] and increases transmissibility of HIV from persons co-infected with HSV-2 and HIV through increasing plasma HIV-1 RNA [15]; [16] [17]. The relationship between HSV-2 and HIV infection has been extensively studied in sub-Saharan Africa [12]; [13]; [14], but there have been relatively few studies of the relationships between HSV-2 and HIV in the US [18]; [19]; [20].

## Methods

The data reported here are derived from ongoing analyses of data collected from drug users entering the Beth Israel Medical Center drug detoxification and methadone maintenance programs in New York City. The methods for this “Risk Factors” study have been previously described in detail [21]; [22] so only a summary will be presented here. The programs are both large (approximately 5000 admissions per year in the detoxification program and approximately 6000 patients participating in methadone treatment at any point in time) and serve New York City as a whole. There were no changes in the requirements for entrance into the program over the time periods for the data presented here.

Both injecting and non-injecting drug users entering the detoxification and methadone maintenance programs are eligible to participate in the study. The present analyses include only persons who reported that they have never injected illicit drugs (never-injecting drug users or NIDUs). Hospital records and the questionnaire results are checked for consistency on route of drug administration and subjects are examined for physical evidence of injecting. The data presented here are from subjects who participated in the study from 1995 to 1999 and from 2005 to 2011. Changes in the aims of the study did not allow for sampling of never-injecting drug users between 2000 and 2004.

In the detoxification program, research staff visited the general admission wards of the program in a preset order and examined all intake records of a specific ward to construct lists of patients admitted within the prior 3 days. All of the patients on the list for the specific ward were then asked to participate in the study. Among patients approached by our interviewers, willingness to participate has been greater than 95%. After all of the patients admitted to a specific ward in the 3-day period have been asked to participate and interviews have been conducted among those who agreed to participate, the interviewer moved to the next ward in the preset order. As there was no relationship between the assignment of patients to wards and the order that the staff rotated through the wards, these procedures should produce an unbiased sample of persons entering the detoxification program. In the methadone program, newly admitted patients (admitted within the previous month) were asked to participate in the research during their intake process. In both programs, willingness to participate has been high, with approximately 95% of those asked agreeing to participate.

Written informed consent was obtained and a structured questionnaire covering demographics, drug use, sexual risk behavior, and use of HIV prevention services was administered by a trained interviewer. Both persons who used drugs through injecting and non-injecting routes of administration were recruited into the study, but the data presented here include only persons who reported never having injected illicit drugs. Most HIV risk behavior questions referred to the 6 months prior to the interview, and thus would cover the time prior to entry into drug treatment. A modest percentage of the male subjects (7% of the 2730 initially interviewed) reported engaging in male-with-male sex. As we were particularly interested in HIV infection due to heterosexual transmission, subjects reporting MSM behavior were not included in the analyses presented here. (A separate report on HSV-2 and HIV among subjects reporting MSM behavior is in preparation.)

After completing the interview, each participant was seen by an HIV counselor for pretest counseling and serum collection. The informed consent specifically included permission to store serum samples for future testing. HIV testing was conducted at the New York City Department of Health Laboratory using a commercial, enzyme-linked, immunosorbent assays (EIA) test with Western blot confirmation (BioRad Genetic Systems HIV-1-2+0 EIA and HIV-1 Western Blot, BioRad Laboratories, Hercules, CA). HSV-2 testing was conducted for all subjects beginning in 2005 and was performed by BioReference Laboratories using the Focus HerpeSelect 1 and 2 ELISA. We used an optical density value of 1.1 or greater for classifying a subject as HSV-2-seropostive on the HerpeSelect assay. Samples positive on HerpeSelect were confirmed using the BioPlex 2200 Multiplex System (Bio-Rad Laboratories, Hercules, CA) We did not test participants surveyed between 1995 and 1999 for HSV-2 at the time of the interview. We were, however, able to assess HSV-2 status by analyzing leftover serum from these participants. Leftover serum was aliquotted into 0.5 ml aliquots, frozen and stored at −70 degrees Celsius. In order to compare relationships between HSV-2 and HIV among NIDUs prior to beginning regular HSV-2 testing in 2005, we selected a stratified sample of 200 NIDUs who participated in the study between 1995 and 1999. Serum samples from these NIDUs were thawed and tested for HSV-1 and HSV-2 as described above. We stratified the sample of NIDUs according to gender and HIV serostatus, such that 100 NIDUs were selected for each gender, with 80 HIV seronegative males, 80 HIV seronegative females, 20 HIV seropositive males and 20 HIV seropositive females. Sampling was random within these strata.

We estimate “population attributable risk percentages” (PAR%s) [23] for HSV-2 infection as a causal factor for increased susceptibility to and increased transmissibility of HIV infection among the female NIDUs. Follwing Freeman et al. [24], we used a Relative Risks of 2 to 3 [12], and the formulas taken from Hennekens &Buring (1987) Epidemiology in Medicine [25]: 1. for increased susceptibility to HIV due to HSV-2 infection among females: 100%×[(Prevalence of HSV-2 among females)×(Relative Risk−1)]/{[(Prevalence of HSV-2 among females)×(Relative Risk-1)]+1}; 2. For increased transmissibility from females to males due to HSV-2/HIV co-infection among females: 100%×[( Prevalence of HSV-2 infection among HIV seropositive females)×(Relative Risk−1)]/{[( Prevalence of HSV-2 infection among HIV seropositive males)×(Relative Risk-1)]+1}. Note that for increased transmissibility the relevant exposure is acquiring HIV from a sexual partner who is HIV seropositive and infected with HSV-2, and non-exposure would be acquiring HIV from a sexual partner who is HIV seropositive but not infected with HSV-2. Following Freeman et al. [12], we used a relative risk of 2.5 for both increased susceptibility and increased transmissibility. The PAR%s for the total sample were the weighted average of the PAR%s by sex.

Stata statistical programs [26] were used for statistical analyses. The study was approved by the Beth Israel Medical Center Institutional Review Board and the National Development and Research Institutes Institutional Review Board.

## Results

### Subject demographics, drug and sexual risk behaviors

Table 1 presents the demographic characteristics, drug use and sexual risk behaviors (during the 6 months prior to interview) for subjects in each of the two time periods (1995–1999 and 2005–2011). Over the two time periods there were significant increases in the percentage of African-Americans, and in the percentage of older (greater than 35 years of age) subjects. There were few changes in sexual risk behaviors, with only a modest increase in subjects reporting multiple sex partners and a modest decrease in subjects reporting unsafe sex with primary partners. There was a significant increase in the percentage of subjects reporting crack cocaine use and a significant decrease in the percentage of subjects reporting intranasal heroin use.

**Table 1:** Demographic and drug use characteristics and sexual risk behaviors among never-injecting drug users, New York City, 1995–99 and 2005–11.

	1995–1999	2005–2011
	n (%)	n (%)
Total	785 (100)	1764 (100)
Gender		
Male	567 (72)	1327 (75)
Female	218 (28)	437 (25)
Race/Ethnicity*		
White	64 (8)	92 (5)
African-American	299 (38)	1185 (67)
Hispanic	404 (52)	442 (25)
Age*		
<35	369 (47)	220 (13)
35 or older	416 (53)	1509 (87)
Multiple Sex Partners*		
No	555 (71)	1121 (64)
Yes	230 (29)	643 (36)
Unsafe sex with primary partner*		
No	317 (40)	985 (56)
Yes	462 (59)	768 (44)
Unsafe sex with casual partner		
No	667 (85)	1432 (82)
Yes	113 (14)	308 (18)
Drug use in past six months*		
Crack cocaine	358 (46)	1281 (73)
Cocaine	362 (46)	726 (41)
Heroin	679 (87)	686 (39)

* significant difference by chi-square test.

### HIV prevalence in 1995–99 and 2005–2011

Table 2 shows HIV prevalence by demographic characteristics and drug use and sexual risk behaviors over the two time periods. HIV prevalence increased from 7% to 13% among the subjects as a whole over the two time periods. There were statistically significant increases in HIV prevalence in almost all demographic and behavioral subgroups over the two time periods. The size of the increases in HIV prevalence—approximate doubling—was quite consistent across the demographic and behavioral subgroups, including the racial/ethnic groups, younger and older subjects, and persons using different drugs. The increase in HIV among the younger subjects indicates that the overall increase was not simply a matter of longer time at risk for the group as a whole. The consistency of the increases across the various subgroups indicates that the overall increase in HIV prevalence (from 7% to 13%) was not due to changes in the demographic composition of the 1995 to 2005–2011 samples.

**Table 2:** HIV seroprevalence among never-injecting drug users, New York City, 1995–1999.

	1995–1999		2005–2011	
	n	HIV positive n (%)	n	HIV positive n (%)
Total	785	55 (7)	1764	237 (13)
Gender				
Male	567	32 (6)*	1327	144 (11)* #
Female	218	23 (11)	437	93 (21)#
Race/Ethnicity				
White	64	1 (2)	92	3 (3)
African-American	299	25 (8)	1185	180 (15)* #
Hispanic	404	29 (7)	442	52 (12)* #
Age				
<35	369	18 (5)	220	23 (11)#
35 or older	416	37 (9)*	1509	207 (14)#
Multiple Sex Partners				
No	555	39 (7)	1121	164 (15)* #
Yes	230	16 (7)	643	71 (11)
Unsafe sex with primary partner				
No	317	36 (11)	985	369 (17)* #
Yes	462	18 (4)*	768	64 (8)#
Unsafe sex with casual partner				
No	667	49 (87)	1432	206 (15)* #
Yes	113	5 (4)	308	25 (8)
Drug use in past six months				
Crack Cocaine	358	34 (10)	1281	207 (16)* #
Cocaine	362	25 (7)	726	67 (9)
Heroin	679	42 (6)	686	59 (9)

# Significant difference by chi square test (comparing same row across time periods).

* Significant difference by chi square test (largest subgroup compared to all others within time period).

### Associations between HSV-2 and HIV

Table 3 shows HSV-2 prevalence by HIV serostatus and sex for the two time periods. There are multiple patterns in these data. First, HSV-2 was extremely high among HIV seropositive females in both time periods (95% and 98%). Second, HSV-2 prevalence was quite high among HIV seronegative females in both time periods (76% and 83%) and high among HIV seropositive males in both time periods (65% and 69%). HSV-2 was significantly higher among HIV seropositives compared to HIV seronegatives, and higher among females compared to males.

**Table 3:** HSV-2 seroprevalence by HIV seroprevalence and by gender among never-injecting drug users, New York City, 1995–1999 and 2005–2011.

	1995–1999	2005–2011
	n+/N (%)	n+/N (%)
HIV negatives total	91/159 (57)#	857/1527 (56)#
HIV negative males	30/79 (38)*	572/1183 (48)*
HIV negative females	61/80 (76)*	285/344 (83)*
HIV positives total	30/37 (81)#	190/237 (80)#
HIV positive males	11/17 (65)*	99/144 (67)*
HIV positive females	19/20 (95)*	91/93 (98)*

* Significant differences for males vs. females by chi-square test.

# Significant differences for HIV seropositives vs. HIV seronegatives by chi-square test.

HSV-2 was significantly associated with HIV in both time periods, odds ratio (OR) = 3.2 (95% CI 1.4 to 7.6) for 1995–1999 and OR = 3.2 (95% CI 2.3 to 4.4) for 2005–2011.

### Patterns of no-infection, mono-infection and co-infection for HSV-2 and HIV

**Figure 1:**
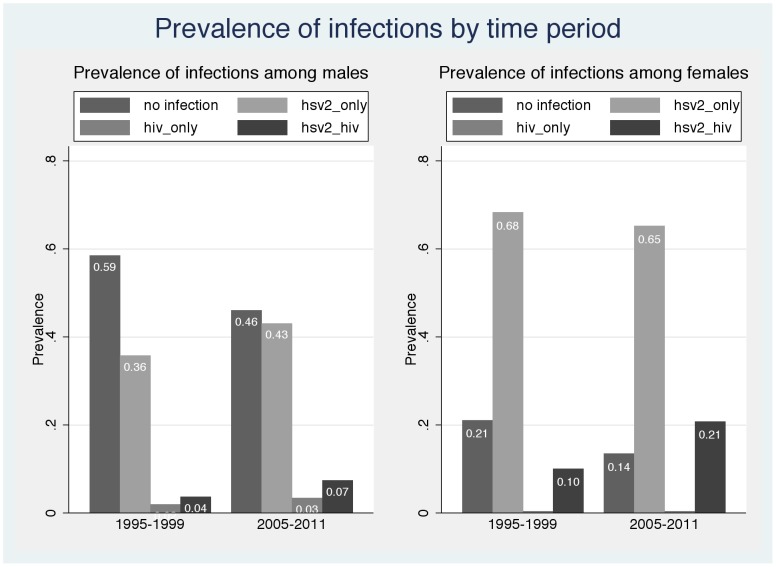
HSV-2 and HIV Infection among non-injecting drug users who participated in Beth Israel Medical Center treatment programs by time period: 1995–199 and 2005–2011.

The extremely high HSV-2 prevalence among the HIV seropositive females, however, suggests that an odds ratio may not accurately capture the relationship between infection with the two viruses. Figure 1 shows the percentages of males and females infected with 1) neither virus, 2) HSV-2 only, 3) HIV only, and 4) both HSV-2 and HIV in each time period. (Note percentages for 1995–1999 are based on weighting the stratified samples that were tested for HSV-2). Among the females, the two most striking aspects of Figure 1 are the relatively low percentages who are not infected with either virus (21% in 1995–1999 and only 14% in 2005–2011) and the extremely low percentages of all females who are infected with HIV only (<0.5% of all females in both time periods).

There was a similar pattern among males, though the pattern was less extreme. A plurality of male subjects were not infected with either virus (59% in 1995–1999 and 46% in 2005–2011). There were very modest percentages of the males who were HIV seropositive but HSV-2 seronegative (2% of all males in 1995–1999 and 3% of all males in 2005–2011).

Among both males and females the major changes over the two time periods were 1) the decreases in the percentage of subjects with no infections and 2) the increases in the percentage of subjects infected with both HSV-2 and HIV (corresponding to the increase in HIV prevalence shown in Table 2).

### Quantitative estimation of the contribution of HSV-2 infection to HIV infection among NIDUs

As noted in the introduction, HSV-2 increases both susceptibility to and transmissibility of HIV. That is, persons who are infected with HSV-2 are at increased risk for becoming infected with HIV, and persons infected with both HSV-2 and HIV are at increased risk of transmitting HIV to others (compared to persons who are infected with HIV but not with HSV-2). Population attributable risk percentages (PAR%s) are a standard method for assessing the importance of a casual risk factor (HSV-2 infection) in the incidence of a disease (HIV infection) in a population. To directly calculate the PAR%s for HSV-2 infection causing increased susceptibility to HIV infection and HSV-2 infection causing increased transmissibility of HIV infection in our population of NIDUs, we would need to know: a) the frequencies of risk behavior (unsafe sex) between HIV seropositive and HIV seronegatives NIDUs so that we could control for risk behavior in assessing the effects of HSV-2 on HIV transmission, b) the HSV-2 status of the NIDUs acquiring HIV when new HIV transmissions occurred, and c) the HSV-2 status of the NIDUs transmitting HIV when new HIV transmissions occurred.

We do not have this information for our population of NIDUs. However, the very high rates and narrow ranges of HSV-2 among female NIDUs permit us to make some reasonable assumptions in order to quantitatively estimate the importance of HSV-2 infection for heterosexual transmission of HIV among the NIDUs. We used the following two assumptions:

1. Since HSV-2 among HIV seronegative female NIDUs was 76% in 1995–1999 and 83% among female NIDUs in 2005–2011, we assumed an average of 80% HSV-2 seroprevalence among female NIDUs when they acquired HIV from male NIDUs.
2. Since HSV-2 among HIV seropositive female NIDUs was 95% in 1995–1999 and 98% among female NIDUs in 2005–2011, we assumed an average of 97% HSV-2 seroprevalence among female NIDUs when they transmitted HIV to male NIDUs.

Freeman et al. conducted a meta-analysis of studies of the effects of HSV-2 on HIV transmission and found that HSV-2 infection creates a relative risk of 2 to 3 for acquisition HIV [12]. (This was the relative risk after controlling for risk behaviors and would represent the increased susceptibility due to the biological effects of HSV-2 on susceptibility to HIV.). In their modeling work on HSV-2 and HIV transmission, Freeman and colleagues [12] also used a relative risk of 2 to 3 for the relative risk of HIV transmission from a person co-infected with HSV-2 and HIV to a sexual partner. We therefore used relative risks of 2 and 3 for estimating PAR%s for increased susceptibility and increased transmissibility of HIV due to HSV-2 infection among our female NIDUs. Table 4 presents the estimated PAR%s for HSV-2 infection causing increased susceptibility to HIV among female NIDUs and increased transmissibility of HIV from female NIDUs. The PAR%s were estimated separately for relative risks of 2 and of 3. (Note formulas used for PAR% estimation given in the methods section.) The estimated PAR%s are quite high, particularly for increased transmissibility of HIV from female NIDUs to their male sexual partners, reflecting the high HSV-2 prevalence among HIV seronegative female NIDUs and the very high HSV-2 seroprevalence among the HIV seropositive female NIDUs.

**Table 4:** Estimated Population Attributable Risk Percentages of HIV infection for HSV-2 as a Cause of HIV Transmission among Female Never-Injecting Drug Users in New York City, 1995–2011.

	PAR %
For RR = 2.PAR% for increased susceptibility for infection from male sexual partners	44%
For RR = 2.PAR% for increased transmissibility to male sexual partners	49%
For RR = 3.PAR% for increased susceptibility for infection from male sexual partners	62%
For RR = 3.PAR% for increased transmissibility to male sexual partners	66%

## Discussion

In this report, we compared two samples of non-injecting drug users entering the Beth Israel Medical Center detoxification and methadone maintenance programs in New York City. There were multiple differences across the two samples; compared to the 1995–1999 subjects, the 2005–2011subjects were older, more likely to be African-American and more likely to smoke crack cocaine. The most dramatic difference, however, was that the 2005–2011 subjects were twice as likely to be HIV seropositive (13%) compared to the 1995–1999 subjects (7%). The doubling in HIV prevalence was quite consistent across demographic and behavioral subgroups, indicating that the increase in HIV prevalence was not a function of differences in the composition of the two samples. The increase in HIV prevalence (6% over a 10 year period) corresponds to an incidence of approximately 1 to 2/100 person-years, which is an unusually high HIV incidence for a heterosexual population in a high income country.

There are undoubtedly multiple factors that contributed to the increase in HIV prevalence over time among these non-injecting drug users. Heterosexual risk behavior is the predominant mode of HIV transmission in this population, (MSM-NIDUs were not included in the analyses), and thus continuing heterosexual risk behavior would be necessary for any increase in HIV prevalence. There was, however, only a very modest increase in sexual risk behavior from 1995–1999 to 2005–2011, so that increased sexual risk behavior is not a likely explanation for the increase in HIV prevalence.

HSV-2 increases both susceptibility to and transmissibility of HIV infection, and the high levels of HSV-2 in the population in both time periods undoubtedly contributed to further spread of HIV among these NIDUs. The estimated PAR%s for increased susceptibility to HIV and increased transmissibility of HIV due to HSV-2 infection among females indicate a major causal role for HSV-2 in HIV infection among these NIDUs. The HSV-2 increases in susceptibility to HIV and increase in transmissibility of HIV for the female NIDUs would be relatively uniform across the racial/ethnic, age and behavior groups in our samples, thus these biological factors could be driving the relatively uniform increases in HIV seen in Table 2. (Note while the increases in HIV were relatively uniform over the two time periods, there are also large differences in HIV prevalence by race/ethnicity and gender in Table 2. These differences are associated with differences in HSV-2 prevalence among the demographic subgroups [27].

HSV-2 prevalence was already high in the 1995–1999 sample, and the increase in HIV prevalence over the two time periods is best seen as a continuation of the effect of HSV-2 infection on HIV transmission that began before 1995 rather than a new development that started in 1995. Indeed, it would be an oversimplification to think of HIV among these NIDUs as only an HIV epidemic. Rather, as shown in Figure 1, essentially all (98%) of the HIV infection among females in both time periods was HSV-2/HIV co-infection and the majority (67%) of HIV infection among males was also HSV-2/HIV co-infection. The rise in HIV among these NIDUs should be considered an epidemic of HSV-2/HIV co-infection.

### Limitations

Several limitations of the present study need to be considered. First, the data presented here are from subjects recruited at a single set of drug treatment programs. However, the treatment programs are very large, serve the city as a whole, and the 2005–2011 data are consistent with HIV prevalence among NIDUs recruited from community settings in New York City in 2004 [9] and with the HIV prevalence among “high risk heterosexuals” in the 2006–2007 National HIV Behavioral Survey conducted in New York City. The NHBS study recruited subjects from high-risk neighborhoods in New York and found an overall HIV prevalence of 8.6% [19]. Our findings are also consistent with modeling work done by Freeman et al. [24] which indicates that the importance of HSV-2 infection in the continuing transmission of HIV increases as an epidemic matures, and that the effect of HSV-2 on increased transmissibility of HIV is more important than the effect of HSV-2 on increased susceptibility.

Second, the research design used in this study was multiple cross sectional surveys, so it was not possible to observe new HSV-2 and new HIV infections in individual subjects. This meant that we were not able to identify the risk behaviors or HSV-2 status of the person acquiring HIV or the risk behaviors or HSV-2 status of the person transmitting HIV at the time of transmission. We thus could not estimate relative risks for the biological effects of HSV-2 infection on susceptibility and transmissibility specific to our population but had to use relative risks taken from the literature. If the biology of HSV-2 infection among our NIDU subjects is greatly different from the biology of HSV-2 infection among females in sub-Saharan Africa, where most of the research on HSV-2 and HIV has been conducted, then our estimates of the PAR%s for female NIDUs would be in error. The very strong relationship between HSV-2 and HIV infection we observed, however, does suggest that HSV-2 does create substantially increased susceptibility to HIV among our female NIDUs.

We would note that conducting a prospective cohort study over decades, with an estimated HIV incidence of 1–2/100 person-years, measuring HSV-2 prevalence in both persons acquiring and transmitting HIV, to determine a statically reliable estimate of the numbers of persons who became infected with HIV, transmitted HIV to others, and then became infected with HSV-2, would be prohibitively expensive.

Because of a substantial minority (one third) of the HIV infected male NIDUs were not infected with HSV-2, we did not estimate PAR%s for the male NIDUs. The very high HSV-2 prevalence among HIV seropositive females (95% in 1995–1999 and 98% in 2005–2011, Table 3 and Figure 1) did permit reasonable estimation of PAR%s for the female NIDUs, which were quite high. Approximately half of the HIV acquisition among female NIDUs may be attributable to HSV-2 increased susceptibility and perhaps over four-fifths of HIV transmission from female NIDUs to male sexual partners may be attributable to HSV-2 increased transmissibility. To the extent that our female NIDUs became infected with HIV and then became infected with HSV-2, our PAR% would overestimate the importance of HSV-2 for HIV acquisition among our subjects. To the extent that our female NIDUs became infected with HIV and transmitted HIV to sexual partners and then became infected with HSV-2, our PAR% for increased transmissibility would overestimate the contribution of HSV-2 to HIV transmission. The data in Figure 1, however, suggest that these would be rare events. Because we did not include consideration of the effects of HSV-2 among the male NIDUs, the PAR%s presented here almost certainly underestimate the contribution of HSV-2 to HIV transmission among these NIDUs.

Third, we did not collect data from non-injecting drug users entering the Beth Israel Medical Center detoxification program between 1999 and 2005. However, blinded HIV testing was conducted on leftover sera from non-injecting drug users entering the Beth Israel Medical Center methadone maintenance treatment program during those years. Among non-injecting drug users entering the methadone program, HIV seroprevalence rose from 9% in 1999 to 14% in 2005, (unpublished data) which is clearly consistent with trend reported here from subjects in this study.

Finally, we were not able to assess sexual mixing between NIDUs and other groups in the city. It is quite likely that some of the HSV-2 and HIV transmission occurred between NIDUs and persons who injected drugs (PWID) and between NIDUs and persons who did not use heroin, cocaine and methamphetamines. However, it does not seem that transmission from other groups was sufficiently common to alter the patterns of HSV-2 and HIV infection seen in both NIDU samples.

These limitations are important, but it is very difficult to imagine how they would have created the strong patterns in HSV-2 and HIV infection and the increase in HIV prevalence over time that we observed. Rather it is much more likely that these patterns are sufficiently strong that we observed them despite the limitations.

## Conclusions

HIV infection among non-injecting drug users in New York City doubled from the late 1990s to the 2000s. While there were undoubtedly multiple causes for this increase, the high prevalence of HSV-2 infection among the male NIDUs and the very high HSV-2 prevalence among female NIDUs indicate that HSV-2 was clearly a major factor. The change in HIV prevalence is best described as a continuation of an epidemic of HSV-2/HIV co-infection that was already underway by 1995.

There is a clear need to implement interventions to reduce HSV-2 related HIV transmission among non-injecting drug users and their sexual partners. There are a number of psychosocial/behavioral interventions to reduce unsafe sexual behavior among injecting and non-injecting drug users, but these have typically demonstrated only modest effect sizes [28]. There are therapies that suppress HSV-2 infection and reduce the frequency of outbreaks [29], and HSV-2 suppressive therapy may reduce HIV viremia [30]. However, in studies conducted to date, suppressive therapy for HSV-2 did not reduce the transmission of HIV [17]. Additional research, including studying higher dosages of suppressive therapy would be useful. HSV-2 suppressive therapy prior to ART and/or in combination with ART should also be investigated, as a recent study found evidence of HIV in the semen of men who had reached viral suppression on ART [31].

HIV/HSV-2 co-infected persons would appear to be high priority for “Treatment as Prevention,” providing anti-retroviral therapy (ART) to HIV-infected persons at all CD4 cell counts in order to reduce HIV transmission to their sexual partners [32]. This should provide benefits to both the HIV infected person and—through reducing transmission—to the community as a whole. The New York City Department of Health and Mental Hygiene announced a new policy in December, 2011 that ART should be offered to all HIV seropositive persons in the city regardless of CD4 cell count [33]. The US Preventive Services Task Force has recommended ART for all HIV seropositive persons in the country [34]. HIV/HSV-2 co-infected persons with substance use disorders may require additional support services in order to initiate and adhere to ART, however, given their increased transmissibility for HIV, the additional costs for providing the needed support services would be fully justified.
